# Longissimus dorsi transcriptome analysis of purebred and crossbred Iberian pigs differing in muscle characteristics

**DOI:** 10.1186/1471-2164-15-413

**Published:** 2014-05-31

**Authors:** Cristina Óvilo, Rita Benítez, Almudena Fernández, Yolanda Núñez, Miriam Ayuso, Ana Isabel Fernández, Carmen Rodríguez, Beatriz Isabel, Ana Isabel Rey, Clemente López-Bote, Luis Silió

**Affiliations:** Dpto Mejora Genética Animal, INIA, Ctra Coruña km 7.5, Madrid, 28040 Spain; Dpto Producción Animal, Facultad de Veterinaria, UCM, Madrid, Spain

**Keywords:** Iberian pig, Transcriptome, Genetic type, Transcription factors, Growth, Meat quality, Metabolism

## Abstract

**Background:**

The two main genetic types in Iberian pig production show important phenotypic differences in growth, fattening and tissue composition since early developmental stages. The objective of this work was the evaluation of muscle transcriptome profile in piglets of both genetic types, in order to identify genes, pathways and regulatory factors responsible for their phenotypic differences. Contemporary families coming from pure Iberian pigs (IB) or from crossing with Duroc boars (DU×IB) were generated. Piglets (14 from each genetic type) were slaughtered at weaning (28 days) and *longissimus dorsi* was sampled for composition and gene expression studies. RNA was obtained and hybridized to Affymetrix *Porcine Genechip* expression arrays.

**Results:**

Loin muscle chemical composition showed significant differences between genetic types in intramuscular fat content (6.1% vs. 4.3% in IB and DUxIB animals, respectively, *P* = 0.009) and in saturated (*P* = 0.019) and monounsaturated fatty acid proportions (*P* = 0.044). The statistical analysis of gene expression data allowed the identification of 256 differentially expressed (DE) genes between genetic types (FDR < 0.10), 102 upregulated in IB and 154 upregulated in DU×IB. Transcript differences were validated for a subset of DE genes by qPCR. We observed alteration in biological functions related to extracellular matrix function and organization, cellular adhesion, muscle growth, lipid metabolism and proteolysis. Candidate genes with known effects on muscle growth were found among the DE genes upregulated in DU×IB. Genes related to lipid metabolism and proteolysis were found among those upregulated in IB. Regulatory factors (RF) potentially involved in the expression differences were identified by calculating the *regulatory impact factors*. Twenty-nine RF were found, some of them with known relationship with tissue development (*MSTN*, *SIX*4, *IRX3*), adipogenesis (*CEBPD, PPARGC1B*), or extracellular matrix processes (*MAX*, *MXI1*). Correlation among the expression of these RF and DE genes show relevant differences between genetic types.

**Conclusion:**

These results provide valuable information about genetic mechanisms determining the phenotypic differences on growth and meat quality between the genetic types studied, mainly related to the development and function of the extracellular matrix and also to some metabolic processes as proteolysis and lipid metabolism. Transcription factors and regulatory mechanisms are proposed for these altered biological functions.

**Electronic supplementary material:**

The online version of this article (doi:10.1186/1471-2164-15-413) contains supplementary material, which is available to authorized users.

## Background

Intramuscular fat (IMF) content and fatty acid (FA) composition are critical aspects of pig meat because of their influence on the sensorial and technological aspects of meat quality such as juiciness, flavor, tenderness and overall desirability of meat [1, 2]. Consequently, research on muscle lipid deposition is currently one of the most important fields of study in meat science [3]. Recently, the research interest on this topic includes the complex physiological and genetic mechanisms of IMF deposition and gene expression patterns and interactions along development [4].

Within skeletal muscle, lipids are stored as droplets both in myofiber cytoplasm and in adipocytes (interspersed between fiber fasciculi) with IMF deposition being highly dependent on the number of intramuscular adipocytes [5]. Although adipocyte differentiation starts early in fetal stages, the increase in adipose cell number and size is maintained along early postnatal growth and later development [6]. IMF is considered a late-developing depot because hyperplasia and not only hypertrophy occurs postnatally, as demonstrated in both pigs and cattle [6, 7]; and hypertrophy by lipid filling of adipocytes persists in late stages [8]. Nevertheless, regulation of intramuscular adipocyte differentiation and growth and triglyceride storage is not completely understood and previous studies suggest that preadipocyte differentiation and lipogenesis exhibit breed-related scheduling [9]. Among different breeds, the adipocyte depots can be regulated differently, and may have a different propensity to metabolize lipids.

Comparison of transcriptome of skeletal muscles and other tissues between phenotypically different pig breeds has been proposed to improve the understanding of the genetic mechanisms underlying differences in growth and meat quality [10]. Several transcriptome studies have compared lean (Landrace, Large-White) vs. fatty breeds (mainly Chinese and other local obese breeds) [11–16]. Also the Duroc breed has been compared to the Pietrain lean breed in prenatal stages [17, 18] and to the Chinese obese Taoyuan breed [19]. Some of these previous works have been focused on the study of myogenesis and thus different stages of fetal development were studied, since myogenesis is assumed to be a predominantly prenatal process [20]. Nevertheless, recent work has shown that hyperplastic muscle growth also occurs from birth to weaning in pigs [21].

The Iberian pig breed is a fatty breed with clear differences in growth rate, adipogenic potential and meat quality with respect to many other porcine breeds [22]. Meat and dry-cured Iberian pig products, characterized by its high prices, come from two main genetic types: purebred Iberian and crossbred Iberian with Duroc boars. In the crossbred animals, the growth performance and primal cuts yield are improved, but conversely, meat quality decreases because their muscles contain lower proportions of IMF and monounsaturated fatty acids (MUFA), as oleic acid [23]. Phenotypic differences between both types in lean and adipose tissue growth are visible from early developmental stages [24]. Regarding the analysis of the Iberian pig transcriptome, a comparison with those of Large-White and other breeds for several tissues at 3 m of age has been reported [25, 26], but no comparison has been performed on the muscle transcriptome between the Iberian and Duroc breeds or their crosses.

Our objective was to evaluate the muscle genome expression profiles of Iberian and Duroc × Iberian genetic types in order to identify the genes and molecular pathways involved in their phenotypic differences. In contrast to previous studies with extreme breeds, the genetic types here compared share the same production system, being genetically closer and phenotypically more similar. We selected the *longissimus dorsi* muscle because it is a prime cut of high economic relevance for fresh and cured pork production. Muscle transcriptome was studied at weaning (28d), as this developmental stage is highly proliferative and relevant for the differentiation of muscular and adipose cells. Additionally, transcriptome information was employed for the identification of transcriptional regulators potentially involved in the different gene expression profiles observed in both genetic types.

## Results and discussion

### Phenotypic differences between genetic types

At weaning, 28 male piglets (14 of each genetic type) were slaughtered and loin muscle was sampled for composition and gene expression studies. Mean live weight at slaughter was 8.03 kg (SD = 1.59 kg). There was no significant difference in live weight between both genetic types. The percentage of loin IMF was higher in purebred Iberian than in crossbred animals (*P* = 0.009, Table 1). Differences in muscle fatty acid composition were also observed, with a higher MUFA content in IB and a higher saturated FA content in DU×IB (Table 1). These results confirm the differential trend in fattening and meat quality traits which is evident since this very early growth stage.

**Table 1:** **Live weight and** ***Longissimus dorsi*** **muscle fat and fatty acid content in IB and DU×IB piglets at weaning (28d)**

Trait	Iberian (n = 14)	Duroc × Iberian (n = 14)	***P*** value
	Mean ± SEM	Mean ± SEM	
Live weight (kg)	8.22 ± 0.37	7.85 ± 0.54	0.567
Intramuscular fat (g/100 g fresh tissue)	6.07 ± 0.45	4.27 ± 0.43	0.009
C16:0, % palmitic acid	23.15 ± 0.66	24.95 ± 0.64	0.051
C18:0, % estearic acid	8.03 ± 0.25	9.23 ± 0.24	0.001
C18:1n-9, % oleic acid	38.64 ± 1.28	37.05 ± 1.25	0.362
C18:2n-6, % linoleic acid	14.36 ± 0.60	15.75 ± 0.68	0.095
SFA, % saturated fatty acids	33.20 ± 0.88	36.17 ± 0.86	0.019
MUFA, % monounsaturated fatty acids	47.81 ± 1.55	43.42 ± 1.51	0.044
PUFA, % polyunsaturated fatty acids	18.97 ± 0.92	20.40 ± 0.90	0.259

### Transcriptome study: identification and functional characterization of differentially expressed genes associated with pure Iberian or crossbred genetic types

Among the platforms currently available, the Affymetrix Porcine array is the most sensitive and reproducible microarray for swine genomic studies [27]. Employing this platform, we detected 271 differentially expressed (DE) probes according to genetic type, exceeding the threshold *PP*-value corresponding to a FDR < 0.10 (*Posterior Probability* =0.006) (Additional file 1). These correspond to 256 known genes. Ten DE genes were represented by more than one DE probe (*CASQ2, ERO1L, IGF2, MTUS2, LOX, MAP1B, ME1, PTPRD, SORT1, SVIP*). Out of the 256 DE genes, 154 were overexpressed in DU×IB and 102 were overexpressed in IB. Regarding the size of the effects, the DE genes upregulated in DU×IB ranged between 1.23 and 5.98 fold-changes, and the ones upregulated in IB ranged between 1.21 and 7.87.

Real-time quantitative PCR (qPCR) was employed to assess the expression of 18 genes (eight upregulated in DU×IB, eight upregulated in IB and two unchanged ones), selected to represent different magnitudes of the differential expression detected in the microarray study. Twelve out of the 16 DE genes and one coming from the non-DE group were successfully validated as DE; and two out of the four non-validated genes (*PLA1A* and *CASP4*) were close to statistical significance (Table 2). The correlations among gene expression values obtained with microarray and qPCR were significant in 15 out of the 18 selected genes (Table 2). Overall, qPCR results were in the same direction and similar magnitude compared to the microarray. The *ELOVL6* gene showed the lowest agreement between methods, which could be due to the detection of different splice variants, as up to 13 different transcripts have been described for this gene in humans. Interestingly, the *SCD* gene, which was selected as a control non-DE (1.5× higher expression in IB, but without statistical significance), was observed to be significantly DE in the qPCR validation step (2× upregulation in IB, *P* = 0.03). The substantial value of Concordance Correlation Coefficient calculated between microarray and qPCR fold-change values for these 18 genes (CCC = 0.863) indicates a remarkable agreement between both measures, validating the global reproducibility of microarray results [28].

**Table 2:** **Technical validation of microarray results by qPCR**

Gene	Ratio microarray (DU×IB)/IB	***PP value*** microarray	ratio qPCR (DU×IB)/IB	***P value*** qPCR	Correlation ( ***r*** )	***P value (H***_***o***_ ***:r = 0)***
*IGF2*	4.88	<0.00001	2.95	0.0001	0.796	<0.0001
*KERA*	3.41	0.00001	2.41	0.0009	0.889	<0.0001
*FMOD*	3.35	0.00006	2.78	0.0048	0.764	<0.0001
*COL1A1*	2.18	0.00082	2.15	0.0060	0.540	0.0030
*FBN2*	1.88	0.00009	1.76	0.012	0.744	<0.0001
*AEBP1*	1.81	0.00111	2.00	0.019	0.832	<0.0001
*LOX*	1.72	0.00071	1.58	0.019	0.506	0.0084
*FKBP14*	1.72	0.00005	1.60	0.025	0.794	<0.0001
*PSMD11*	0.73	0.00541	0.65	0.046	0.444	0.0229
*ALOX5AP*	0.70	0.00139	0.78	0.128	0.189	0.3331
*CASP4*	0.64	0.00216	0.54	0.084	0.869	<0.0001
*ELOVL6*	0.64	0.00114	1.11	0.561	0.086	0.6616
*NFKBIZ*	0.59	0.00369	0.82	0.358	0.502	0.0065
*ME1*	0.50	0.00028	0.57	0.024	0.802	<0.0001
*PLA1A*	0.42	0.00017	0.42	0.074	0.769	<0.0001
*PON3*	0.31	<0.00001	0.37	0.001	0.787	<0.0001
*SCD*	0.65	0.15186	0.50	0.030	0.866	<0.0001
*ELOVL5*	1.07	0.28406	1.13	0.433	0.255	0.2089

Functions and pathways altered by genetic type were explored by studying overrepresentation of gene ontology (GO) terms on the three main family categories with DAVID tool (Database for Annotation, Visualization and Integrated Discovery) [29] (Additional file 2). Most enriched terms were related to main biological processes including: extracellular structure organization, developmental process, lipid metabolic process and muscle organ development. Regarding the cellular compartment GO category, the extracellular matrix (ECM) part was highly enriched in the list of DE genes.

The lists of genes upregulated in IB and DU×IB were also separately explored (Tables 3 and 4). In order to reduce the redundancy, a functional annotation clustering was performed with DAVID tool. Genes upregulated in IB affected GO terms grouped in three main annotation clusters, which are related to lipid metabolic process, transcriptional regulation and proteolysis (Table 3). Genes upregulated in DU×IB affected biological process terms clustered in annotation groups related to development, ECM organization, response to stimulus and cell migration and proliferation (Table 4). DAVID tool also allowed for the identification of KEGG pathways significantly enriched in the list of genes upregulated in each genetic type (Additional file 2). *Ubiquitin mediated proteolysis* was significantly enriched in IB, while several KEGG pathways were overrepresented in the DU×IB type. Among them the most significant ones were *ECM-receptor interaction* and *Focal adhesion*.

**Table 3:** **Functional annotation clustering of genes upregulated in muscle from Iberian piglets**

**Annotation Cluster 1 Enrichment Score: 2.33**	**Count**	***P*** **value** ^**+**^	**Genes**
GO:0044255 ~ cellular lipid metabolic process	11	0.00007	PLAA, PTGES3, SAMD8, AGPAT5, ALOX5AP, SCD, EPHX2, PLA1A, GNPAT, ELOVL6, SIRT1
GO:0006644 ~ phospholipid metabolic process	4	0.034	PLAA, SAMD8, AGPAT5, PLA1A
GO:0019637 ~ organophosphate metabolic process	4	0.037	PLAA, SAMD8, AGPAT5, PLA1A
**Annotation Cluster 2 Enrichment Score: 2.11**	**Count**	***P*** **value** ^**+**^	**Genes**
GO:0006350 ~ transcription	16	0.001	NFKBIZ, ZFP30, RBM4, AFF4, AFF3, ZNF143, SIRT1, CBFB, MAX, CNTF, ZNF326, IRF1, PHTF1, NFE2L1, DNTTIP2, MLLT3
GO:0006366 ~ transcription from RNA polymerase II promoter	5	0.001	MAX, AFF4, IRF1, NFE2L1, CBFB
GO:0032774 ~ RNA biosynthetic process	5	0.005	MAX, AFF4, IRF1, NFE2L1, CBFB
GO:0006351 ~ transcription, DNA-dependent	5	0.005	MAX, AFF4, IRF1, NFE2L1, CBFB
GO:0045449 ~ regulation of transcription	19	0.007	NFKBIZ, ZFP30, RBM4, AFF4, AFF3, ZNF143, SIRT1, CBFB, MAX, CNTF, NEDD4, ZNF326, AGT, TIAL1, IRF1, PHTF1, NFE2L1, DNTTIP2, MLLT3
**Annotation Cluster 3 Enrichment Score: 1.69**	**Count**	***P*** **value** ^**+**^	**Genes**
GO:0006508 ~ proteolysis	13	0.003	CTSL2, ASB11, MYLIP, C4BPA, UBE2QL1, MARCH6, UBE2D4, CASP4, PSMD11, NEDD4, KLHL15, CASP7, RNF19B
GO:0019941 ~ modification-dependent protein catabolic process	9	0.004	UBE2D4, NEDD4, PSMD11, KLHL15, ASB11, RNF19B, UBE2QL1, MYLIP, MARCH6
GO:0043632 ~ modification-dependent macromolecule catabolic process	9	0.004	UBE2D4, NEDD4, PSMD11, KLHL15, ASB11, RNF19B, UBE2QL1, MYLIP, MARCH6
GO:0051603 ~ proteolysis involved in cellular protein catabolic process	9	0.005	UBE2D4, NEDD4, PSMD11, KLHL15, ASB11, RNF19B, UBE2QL1, MYLIP, MARCH6
GO:0044257 ~ cellular protein catabolic process	9	0.005	UBE2D4, NEDD4, PSMD11, KLHL15, ASB11, RNF19B, UBE2QL1, MYLIP, MARCH6
GO:0030163 ~ protein catabolic process	9	0.007	UBE2D4, NEDD4, PSMD11, KLHL15, ASB11, RNF19B, UBE2QL1, MYLIP, MARCH6
GO:0009057 ~ macromolecule catabolic process	10	0.008	NGLY1, UBE2D4, NEDD4, PSMD11, KLHL15, ASB11, RNF19B, UBE2QL1, MYLIP, MARCH6
GO:0044265 ~ cellular macromolecule catabolic process	9	0.014	UBE2D4, NEDD4, PSMD11, KLHL15, ASB11, RNF19B, UBE2QL1, MYLIP, MARCH6

^+^ *P*-value from modified Fisher exact score.

**Table 4:** **Functional annotation clustering of genes upregulated in muscle from DU×IB piglets**

Annotation Cluster 1 Enrichment Score: 6.94	Count	***P*** value^+^	Genes
GO:0032501 ~ multicellular organismal process	54	6.61E-13	S100A6, AEBP1, KERA, UTRN, POSTN, REST, ENPEP, GPX2, APP, ROBO1, SEMA3E, S1PR5, COL12A1, LOX, SLC22A2, USH2A, EGFL6, CHODL, CTNNBIP1, MAN2A1, ALDH7A1, DACT1, GRN, SORT1, VCAN, COL1A1, CNTN3, PROS1, CASQ2, MYL6, HUS1, AKAP9, GPM6B, FKBP1A, APLNR, PTK2, ITGAV, SYN3, PPP3CB, SCARB1, FBN2, DCLK1, MAP1B, ITGA2, IGF2, COL5A1, DKK3, ITGA6, SFRP2, CD59, TCF12, MYH10, CDH11, CLCN5
GO:0048856 ~ anatomical structure development	41	9.60E-10	MYL6, AEBP1, S100A6, UTRN, GPM6B, POSTN, FKBP1A, ENPEP, REST, PTK2, APP, ROBO1, ITGAV, S1PR5, SEMA3E, PPP3CB, COL12A1, FBN2, LOX, USH2A, DCLK1, MYOC, MAP1B, CHODL, ITGA2, IGF2, COL5A1, DKK3, MAN2A1, ITGA6, SFRP2, GRN, SORT1, VCAN, ANTXR1, CNTN3, COL1A1, TCF12, CASQ2, MYH10, CDH11
GO:0007275 ~ multicellular organismal development	43	2.42E-09	MYL6, AEBP1, S100A6, HUS1, UTRN, GPM6B, POSTN, FKBP1A, ENPEP, REST, PTK2, APP, ROBO1, ITGAV, S1PR5, SEMA3E, PPP3CB, COL12A1, FBN2, LOX, USH2A, DCLK1, EGFL6, MAP1B, CHODL, ITGA2, IGF2, COL5A1, CTNNBIP1, DKK3, MAN2A1, DACT1, ITGA6, SFRP2, GRN, SORT1, VCAN, CNTN3, COL1A1, TCF12, CASQ2, MYH10, CDH11
GO:0032502 ~ developmental process	45	4.28E-09	MYL6, S100A6, AEBP1, HUS1, UTRN, GPM6B, POSTN, FKBP1A, ENPEP, REST, PTK2, APP, ROBO1, ITGAV, S1PR5, SEMA3E, PPP3CB, COL12A1, FBN2, LOX, USH2A, DCLK1, MYOC, EGFL6, MAP1B, CHODL, ITGA2, IGF2, COL5A1, CTNNBIP1, DKK3, MAN2A1, DACT1, ITGA6, SFRP2, GRN, SORT1, VCAN, ANTXR1, CNTN3, COL1A1, TCF12, CASQ2, MYH10, CDH11
GO:0048731 ~ system development	35	1.36E-07	MYL6, AEBP1, S100A6, UTRN, GPM6B, POSTN, FKBP1A, ENPEP, REST, APP, PTK2, ROBO1, ITGAV, S1PR5, SEMA3E, PPP3CB, COL12A1, LOX, USH2A, DCLK1, MAP1B, CHODL, ITGA2, IGF2, COL5A1, MAN2A1, ITGA6, SORT1, VCAN, CNTN3, COL1A1, TCF12, CASQ2, CDH11, MYH10
GO:0030154 ~ cell differentiation	22	2.12E-04	S100A6, EGFL6, UTRN, MAP1B, ITGA2, IGF2, GPM6B, REST, PTK2, APP, ITGA6, ROBO1, SFRP2, SEMA3E, S1PR5, PPP3CB, SORT1, VCAN, ANTXR1, COL1A1, DCLK1, MYH10
GO:0048869 ~ cellular developmental process	22	3.88E-04	S100A6, EGFL6, UTRN, MAP1B, ITGA2, IGF2, GPM6B, REST, PTK2, APP, ITGA6, ROBO1, SFRP2, SEMA3E, S1PR5, PPP3CB, SORT1, VCAN, ANTXR1, COL1A1, DCLK1, MYH10
GO:0048513 ~ organ development	24	4.09E-04	MYL6, AEBP1, UTRN, ITGA2, CHODL, FKBP1A, IGF2, POSTN, ENPEP, COL5A1, MAN2A1, PTK2, APP, ITGA6, ROBO1, ITGAV, PPP3CB, COL1A1, LOX, TCF12, CASQ2, USH2A, DCLK1, MYH10
**Annotation Cluster 2 Enrichment Score: 4.70**	**Count**	***P*** **value** ^**+**^	**Genes**
GO:0043062 ~ extracellular structure organization	11	7.37E-08	APP, PTK2, COL14A1, UTRN, MAP1B, COL12A1, FKBP1A, POSTN, COL1A1, LOX, COL5A1
GO:0030198 ~ extracellular matrix organization	9	5.04E-07	APP, PTK2, COL14A1, COL12A1, FKBP1A, POSTN, COL1A1, LOX, COL5A1
GO:0030199 ~ collagen fibril organization	5	7.49E-05	COL14A1, COL12A1, COL1A1, LOX, COL5A1
**Annotation Cluster 3 Enrichment Score: 2.37**	**Count**	***P*** **value** ^**+**^	**Genes**
GO:0009605 ~ response to external stimulus	15	1.56E-04	MAP1B, ITGA2, IGF2, ENSA, COL5A1, ITGA6, SFRP2, ROBO1, CD59, SCARB1, VCAN, LOX, COL1A1, PROS1, MYH10
GO:0042060 ~ wound healing	8	2.23E-04	CD59, ITGA2, SCARB1, IGF2, LOX, PROS1, COL5A1, MYH10
GO:0009611 ~ response to wounding	10	0.0011093	CD59, MAP1B, ITGA2, VCAN, SCARB1, IGF2, LOX, PROS1, COL5A1, MYH10
GO:0051128 ~ regulation of cellular component organization	9	0.011086	PTK2, ROBO1, MAP1B, ITGA2, SCARB1, IGF2, COL5A1, MYH10, DSTN
GO:0050896 ~ response to stimulus	28	0.0134512	KERA, HUS1, TIPIN, FKBP1A, ENSA, GPX2, APP, ROBO1, PPP3CB, SCARB1, GNG2, LOX, FAM129A, USH2A, MAP1B, ITGA2, IGF2, COL5A1, ABCG2, ITGA6, SFRP2, CD59, SORT1, VCAN, COL1A1, TCF12, PROS1, MYH10
GO:0042221 ~ response to chemical stimulus	14	0.0284588	MAP1B, ITGA2, FKBP1A, IGF2, ENSA, ABCG2, GPX2, ROBO1, PPP3CB, SORT1, SCARB1, GNG2, LOX, COL1A1
**Annotation Cluster 4 Enrichment Score: 2.26**	**Count**	***P*** **value** ^**+**^	**Genes**
GO:0006928 ~ cell motion	13	1.17E-05	ITGA2, ENPEP, COL5A1, DSTN, PTK2, APP, ITGA6, ROBO1, SCARB1, VCAN, DCLK1, THBS4, MYH10
GO:0016477 ~ cell migration	10	1.09E-04	PTK2, ITGA6, ROBO1, VCAN, SCARB1, ENPEP, COL5A1, DCLK1, MYH10, THBS4
GO:0007409 ~ axonogenesis	8	1.21E-04	APP, S100A6, PTK2, ROBO1, MAP1B, VCAN, DCLK1, MYH10
GO:0000904 ~ cell morphogenesis involved in differentiation	9	1.61E-04	APP, S100A6, PTK2, ROBO1, MAP1B, VCAN, ANTXR1, DCLK1, MYH10
GO:0048870 ~ cell motility	10	1.61E-04	PTK2, ITGA6, ROBO1, VCAN, SCARB1, ENPEP, COL5A1, DCLK1, MYH10, THBS4
GO:0051674 ~ localization of cell	10	1.64E-04	PTK2, ITGA6, ROBO1, VCAN, SCARB1, ENPEP, COL5A1, DCLK1, MYH10, THBS4
GO:0030154 ~ cell differentiation	22	2.12E-04	S100A6, EGFL6, UTRN, MAP1B, ITGA2, IGF2, GPM6B, REST, PTK2, APP, ITGA6, ROBO1, SFRP2, SEMA3E, S1PR5, PPP3CB, SORT1, VCAN, ANTXR1, COL1A1, DCLK1, MYH10
GO:0009653 ~ anatomical structure morphogenesis	20	2.24E-04	S100A6, MAP1B, ITGA2, FKBP1A, IGF2, ENPEP, COL5A1, DKK3, PTK2, APP, ITGA6, ROBO1, SFRP2, VCAN, ANTXR1, FBN2, COL1A1, MYOC, DCLK1, MYH10
GO:0048869 ~ cellular developmental process	22	3.88E-04	S100A6, EGFL6, UTRN, MAP1B, ITGA2, IGF2, GPM6B, REST, PTK2, APP, ITGA6, ROBO1, SFRP2, SEMA3E, S1PR5, PPP3CB, SORT1, VCAN, ANTXR1, COL1A1, DCLK1, MYH10
GO:0030030 ~ cell projection organization	10	5.27E-04	APP, S100A6, PTK2, ITGA6, ROBO1, MAP1B, VCAN, DCLK1, MYH10, THBS4
GO:0040011 ~ locomotion	10	5.63E-04	PTK2, ITGA6, ROBO1, VCAN, SCARB1, ENPEP, COL5A1, DCLK1, MYH10, THBS4
GO:0048858 ~ cell projection morphogenesis	8	5.68E-04	APP, S100A6, PTK2, ROBO1, MAP1B, VCAN, DCLK1, MYH10
GO:0032990 ~ cell part morphogenesis	8	9.16E-04	APP, S100A6, PTK2, ROBO1, MAP1B, VCAN, DCLK1, MYH10
GO:0000902 ~ cell morphogenesis	9	0.0013059	APP, S100A6, PTK2, ROBO1, MAP1B, VCAN, ANTXR1, DCLK1, MYH10
GO:0048699 ~ generation of neurons	10	0.0021025	APP, S100A6, PTK2, ROBO1, S1PR5, MAP1B, VCAN, REST, DCLK1, MYH10
GO:0048666 ~ neuron development	8	0.0025921	APP, S100A6, PTK2, ROBO1, MAP1B, VCAN, DCLK1, MYH10
GO:0022008 ~ neurogenesis	10	0.0031988	APP, S100A6, PTK2, ROBO1, S1PR5, MAP1B, VCAN, REST, DCLK1, MYH10
GO:0032989 ~ cellular component morphogenesis	9	0.0032876	APP, S100A6, PTK2, ROBO1, MAP1B, VCAN, ANTXR1, DCLK1, MYH10
GO:0045664 ~ regulation of neuron differentiation	5	0.0042345	PTK2, ROBO1, S1PR5, MAP1B, REST
GO:0007399 ~ nervous system development	13	0.0054731	S100A6, MAP1B, GPM6B, REST, PTK2, APP, ROBO1, S1PR5, SEMA3E, VCAN, CNTN3, DCLK1, MYH10
GO:0048468 ~ cell development	11	0.005626	APP, S100A6, PTK2, ROBO1, UTRN, MAP1B, PPP3CB, VCAN, ANTXR1, DCLK1, MYH10
GO:0030182 ~ neuron differentiation	8	0.0057725	APP, S100A6, PTK2, ROBO1, MAP1B, VCAN, DCLK1, MYH10
GO:0060284 ~ regulation of cell development	6	0.0067586	PTK2, ROBO1, S1PR5, MAP1B, IGF2, REST
GO:0050767 ~ regulation of neurogenesis	5	0.0122736	PTK2, ROBO1, S1PR5, MAP1B, REST
GO:0031344 ~ regulation of cell projection organization	4	0.0132242	PTK2, ROBO1, MAP1B, ITGA2
GO:0051960 ~ regulation of nervous system development	5	0.0177634	PTK2, ROBO1, S1PR5, MAP1B, REST
GO:0045595 ~ regulation of cell differentiation	8	0.0186936	PTK2, ROBO1, ITGAV, S1PR5, MAP1B, IGF2, REST, USH2A
GO:0022604 ~ regulation of cell morphogenesis	4	0.037216	PTK2, ROBO1, MAP1B, MYH10
GO:0050793 ~ regulation of developmental process	9	0.0379454	PTK2, ROBO1, ITGAV, S1PR5, MAP1B, IGF2, REST, USH2A, MYH10

^+^ *P*-value from modified Fisher exact score.

**Figure 1:**
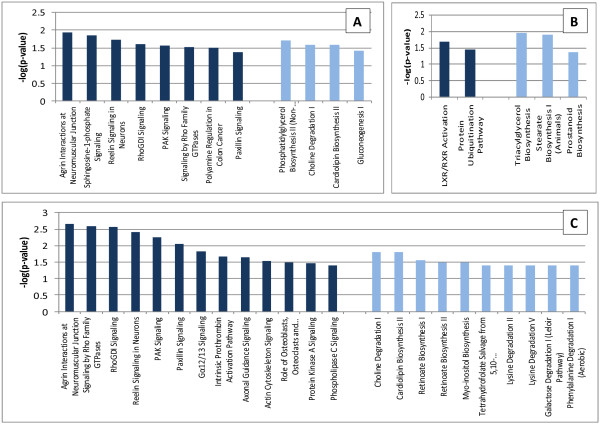
**Functional categorization analysis with IPA.** Canonical pathways significantly enriched in the three sets of genes are shown (*P* < 0.05): **A)** Genes DE between both genetic types; **B)** Genes upregulated in IB; **C)** Genes upregulated in DU×IB. Signaling pathways are indicated with dark bars and metabolic pathways with light bars.

**Figure 2:**
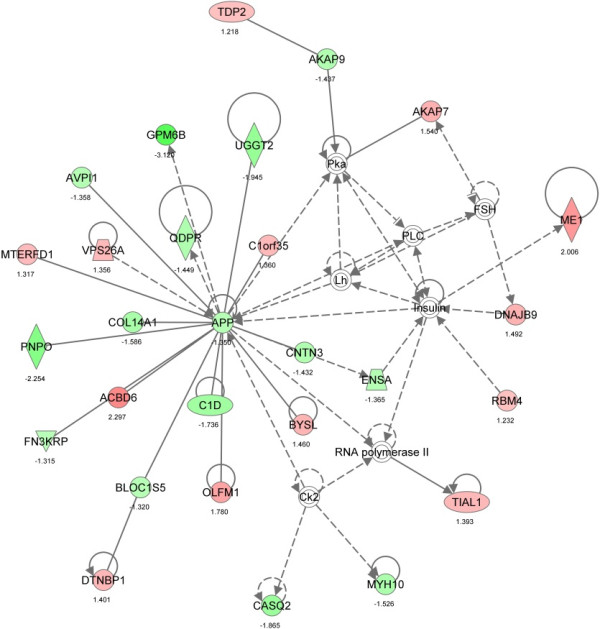
**Gene network 1: Cellular Development, Cellular Growth and Proliferation, Embryonic Development (score 51).** Molecules are represented as nodes and the biological relationships between nodes are represented as edges. Genes upregulated in IB are indicated in red and the ones upregulated in DU×IB are shown in green.

**Figure 3:**
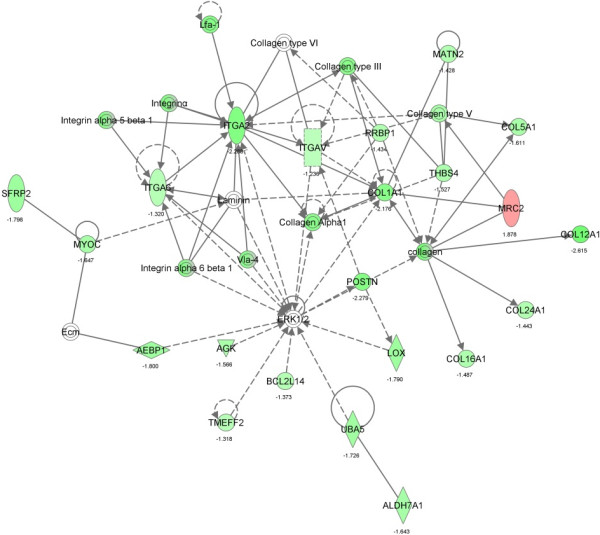
**Gene network 2: Connective Tissue Disorders, Dermatological Diseases and Conditions, Cellular Assembly and Organization (score 47).** Molecules are represented as nodes and the biological relationships between nodes are represented as edges. Genes upregulated in IB are indicated in red and the ones upregulated in DU×IB are shown in green.

**Figure 4:**
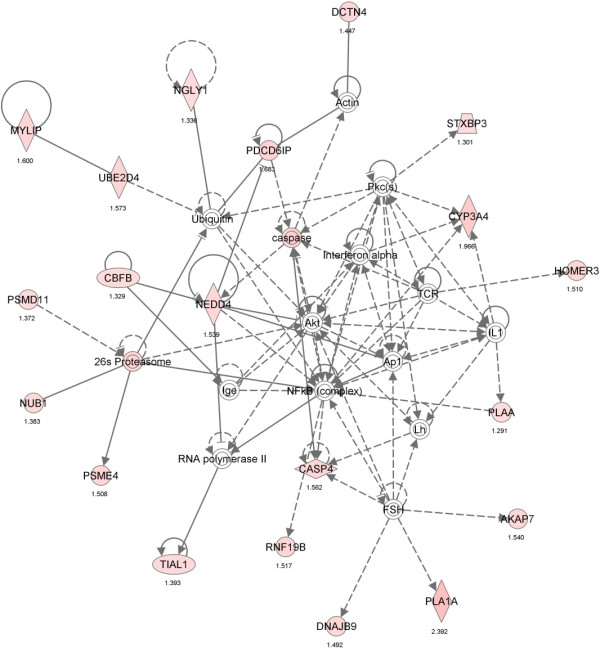
**Gene network 3: Protein Degradation, Protein Synthesis, Cell Morphology (score 41).** Molecules are represented as nodes and the biological relationships between nodes are represented as edges. Genes upregulated in IB are indicated in red and the ones upregulated in DU×IB are shown in green.

The main biological functions identified by Ingenuity Pathway Analysis (IPA) in the comparative dataset of the IB and DU×IB groups included categories related to Cell-To-Cell Signaling and Interaction (*P* = 0.00004, *n* = 35), Lipid Metabolism (*P* = 0.0001, *n* = 43) and Small Molecule Biochemistry (*P* = 0.0001, n = 48). Furthermore, transcripts related to lipid metabolism were mainly upregulated in IB group, while transcripts related to Cell-To-Cell Signaling and Interaction were mainly upregulated in DU×IB group. The specific functions of cell movement/migration and attachment/adhesion of cells were significantly predicted to be decreased in IB samples (*P* = 0.00004 and *P* = 0.0003, respectively). The canonical pathways significantly over-represented in the DE genes are reflected in Figure 1 and the most significant interaction networks (NW) are displayed in Figures 2, 3 and 4. Results concerning the separate analysis of the genes upregulated in each one of the genetic types provide potential specific mechanisms to underlie the biological functions and phenotypic traits changing between them.

The main altered functional patterns observed with the different tools are discussed individually below:

#### Tissue development and ECM organization

Among the DE genes affected by genetic type we found several widely known ones related to muscle development, including *IGF2* (with 5× upregulation in DU×IB), which is the most significant DE gene with seven probes showing differential expression. The growth factor coded by this gene has a major function in muscle promoting fiber differentiation. This locus is paternally imprinted, and a nucleotide substitution in its intron 3 has been described, which abrogates in vitro interaction with a nuclear repressor factor. This substitution affects transcriptional regulation in a way that pigs inheriting the mutation from their sire have a threefold increase in *IGF2* messenger RNA expression in postnatal muscle [30]. This mutation is absent in Iberian pig populations and at very high frequency in the Duroc sire lines employed for crossing with Iberian pigs. In fact, our animals were genotyped for this polymorphism and all DU×IB piglets showed the inheritance of the mutant allele from their Duroc sire, in agreement with the differences observed in gene expression.

We also found other DE genes with roles on myogenesis or muscle development as amyloid beta precursor protein (*APP)* and Fibrillin-2 (*FBN2)*. The *APP* gene has a central role in the most significant gene network detected in this work (Figure 2), related to tissue development. The *APP* appears to promote cell adhesion, acting in an integrin-like manner [31]. Evidence of interaction with laminin and collagen provides further evidence of adhesion-promoting properties. Also studies suggest that peptides derived from the amyloid precursor protein can promote transcriptional activation and can have growth-promoting properties both before and after birth [32]. In fact, *APP*-deficient mice obtained by gene targeting are lighter in body mass. The *FBN2* gene may influence the formation and maintenance of extracellular microfibrils [33], and it has been proposed to play an important role in muscle development being considered a candidate for muscling traits [14, 34]. Another interesting result is the upregulation in the DU×IB muscles of AE binding protein 1 (*AEBP1)* gene, which encodes a member of the carboxypeptidase A protein family. This protein may function as a transcriptional repressor in adipogenesis and muscle cell differentiation, playing a key role in modulation of in vivo adiposity and regulation of energy balance [35]. This protein downregulates *PTEN*, *PPAR*γ*1* and *LXR*α expression and transcriptional activity [36], and influence intracellular lipid accumulation. It promotes proliferation of preadipocytes and inhibits their differentiation into mature, fat-filled adipocytes [37].

Regarding the functions and pathways affected by genetic type, the most significantly affected biological function, according to all the employed tools, is ECM function and organization, which is narrowly related to tissue development (Additional file 2, Table 4, Figures 1 and 3). Genes involved in cell adhesion and extracellular structures were found upregulated in DU×IB, mainly several *integrin* and *collagen* genes and other interacting molecules such as *POSTN*, *LOX*, *MATN2* or *THBS4* (Figure 3). The extracellular matrix consists of a dynamic mixture of structural and functional macromolecules and serves an important role in tissue and organ morphogenesis and in the maintenance of cell and tissue structure and function [38]. It has been shown to be very relevant in cellular signalling because specific interactions between cells and the ECM have pivotal roles in the regulation of muscle cell proliferation and differentiation [39]. These interactions are mediated by transmembrane molecules, mainly integrins and also other cell-surface-associated components, and lead to a direct or indirect control of cellular activities such as adhesion, migration, differentiation, proliferation, and apoptosis [40]. Proliferation determines the pool of muscle cells available for differentiation and thus it influences the potential for muscle growth. Moreover, the extracellular environment regulates the proliferation and differentiation of mesenchymal stem cells and satellite cells [41], which can follow adipogenic differentiation thus increasing the intramuscular adipocytes, fibrogenic differentiation increasing connective tissue content, and of course myogenic differentiation which increases muscle mass. Thus, function of ECM has relevant influence not only on muscle development but also in future meat quality by affecting intramuscular fat deposition and connective tissue abundance [42].

Integrins function as mechanoreceptors and provide a force-transmitting physical link between the ECM and the cytoskeleton and it is proposed that these proteins also regulate angiogenesis [43]. There is considerable evidence of the close relationship between developing adipocytes and vasculature, and hence the influence of integrins on angiogenesis may have an impact on adipogenesis as well. In fact, adipogenesis and angiogenesis are reciprocally regulated [44]. Three different *Integrin* genes were upregulated in DU×IB (*ITGAV*, *ITGA2* and *ITGA6*), which show central positions in NW2 related to Cellular Assembly and Organization, especially the *ITGA2* gene (Figure 3). *ITGA* is considered a myogenesis inhibitor which has been suggested to delay muscle differentiation, contributing to a later but higher differentiation of muscle fibers in lean breeds [14], in agreement with our findings. *ITGAV* is also related to negative regulation of lipid transport and storage. Integrins are narrowly related to collagens and both types of molecules interact in the maintenance of the extracellular matrix. In fact, the DE gene *ITGA2* encodes the alpha subunit of a transmembrane receptor for collagens and related proteins. Six different collagen genes were upregulated in DU×IB piglets (*COL1A1, COL5A1, COL12A1, COL14A1, COL16A1, COL24A1*). Collagen deposition in intramuscular locations starts in fetal stages, and this deposition is predictive of collagen deposition in intramuscular locations in growing pigs [45, 46]. Several works indicate that IGF system, PPARγ and myostatin may regulate the deposition of collagen in pig intramuscular locations [47, 48]. Muscle collagen content contributes to toughness of meat, influences texture and is associated with growth rate [49]. Moreover, collagen development is negatively related to adipocyte development in the ECM: extensive collagen deposition restricts local fat cell clusters growth, and conversely, removal of collagen stimulates the metabolism and survival of adipocytes [50]. Therefore, the higher collagen gene expression in DU×IB muscle is in agreement with the reported higher lean growth but lower meat tenderness and intramuscular fat content of fattened pigs [24].Canonical pathways upregulated in DU×IB (Figure 1) are narrowly related to the effects on ECM. Main signaling pathways are involved in assembly of actin cytoskeleton, cell adhesion to the extracellular matrix, cell-cell adhesion and cell motility and growth (Rho GTPase signaling, PAK signaling, Paxillin signaling, actin cytoskeleton signaling, Gα12 signaling).

Some of our individual DE genes have been previously reported to be DE between phenotypically different breeds or experimental animals, as *IGF2*[16]. Also, ECM components (mainly collagen genes) and functions have been detected as differential in comparison studies among populations differing in intramuscular fat composition, due to breed or dietary factors [15, 17, 51, 52]. These previous results together with the present ones reinforce the ECM biological importance in determining composition and organization of muscle tissue, as well as its significance in the regulation of IMF deposition.

#### Lipid metabolism and protein catabolism

The higher lipid deposition observed in muscle of IB pigs (Table 1), was coincident with the upregulation of genes related to lipid metabolism in this genetic type (Additional file 2 and Table 3). Most genes were involved in lipid and phospholipid biosynthetic processes (for example *ELOVL6, ME1, PTGES3, AGPAT5, GNPAT*) but also some of them had a role in lipid and fatty acid hydrolysis (*PON3, PLA1A*). This is consistent with previous results showing that fatter animals have higher mRNA levels for both lipogenic and lipolytic enzymes, with net lipid deposition being regulated by their ratio rather than enhancing only one of these pathways [10]. The *SCD* (Stearoyl-CoA desaturase) gene was also identified to be upregulated in IB muscle in the qPCR validation step (Table 2). This gene is highly relevant regarding meat quality because its product catalyzes the biosynthesis of monounsaturated fatty acids from saturated fatty acids. The higher expression of this gene in pure Iberian pig muscle is in agreement with the differences in fatty acid contents observed (Table 1), which constitute the main differential trait regarding tissue composition between the analysed genetic types also in adulthood. IPA analysis also indicated that *Lipid Metabolism* was one of the main biological functions in the comparative dataset. Moreover, the most significant canonical signaling pathway represented by the genes upregulated in IB (Figure 1) was involved in the regulation of lipid metabolism, inflammation, and cholesterol to bile acid catabolism (*LXR/RXR activation*) and the three metabolic pathways detected are related to the biosynthesis of lipids and fatty acids: *Triacylglycerol biosynthesis, Prostanoid biosynthesis* and *Stearate biosynthesis*.

When studying genome-wide transcriptional profile on heterogeneous samples as animal tissues it is necessary to take into account the cellular heterogeneity. Any observed differences may be strongly confounded by differences in cell type compositions between samples [53]. In this sense, results regarding lipid metabolism genes are consistent with an influence of differential cellularity between genetic types. Some of the previously mentioned expression differences are in agreement with an earlier adipocyte development in IB muscles, for instance, the DE observed for *AEBP1* gene suggests a higher content of preadipocytes in DU×IB piglets vs. a higher content of mature adipocytes in IB. Also, the higher expression of *LOX*, collagens and other ECM proteins in crossbred muscles may be related to a higher content of preadipocytes [54]. In order to study the potential contribution of this differential cellularity to the expression differences observed, we performed the qPCR quantification of the expression of the preadipocyte marker *DLK1* gene. This gene is highly expressed in preadipocytes and absent after adipocyte differentiation [55], and it is not present in the Affymetrix array. In spite of its low expression level in muscle, we detected significant DE in the gene according to genetic type. Muscles from crossbred piglets showed 1.5- fold upregulation (*P* < 0.023). This interesting result joint with the other DE genes detected supports the hypothesis of higher content of preadipocytes in crossbred animals’ vs. higher content of mature adipocytes in Iberian piglets, and thus earlier adipogenesis in the last ones. On the other hand, a higher lipogenic capacity in muscle of pure IB animals being reflected at the genetic level could be suggested.

Gene ontology enrichment and clustering analyses show that protein catabolic processes are significantly enriched in the dataset of genes upregulated in IB (Table 3 and Additional file 2). *Ubiquitin mediated proteolysis* KEGG pathway is predicted to be upregulated in IB. Also, according to IPA, the second main canonical pathway affected by the genes upregulated in IB is *Protein ubiquitination* pathway (Figure 1). The protein ubiquitination pathway plays a major role in the degradation of short-lived or regulatory proteins involved in a variety of cellular processes [56]. DE genes affecting protein ubiquitination include *UBE2D4, NEDD4, PSMD11, CUL9, KLHL15, ASB11, RNF19B, UBE2QL1, MYLIP, MARCH6*. Apart from this specific pathway which is modification-dependent, gene ontology analysis allows the detection of other proteolytic enzymes as *C4BPA, CASP4, CASP7* or *CTSL2*. IPA also provides a significant gene NW related to proteolysis (Figure 4), composed exclusively of genes upregulated in IB.

Protein degradation may be the main cause of the poor muscle development of purebred Iberian pigs. According to previously reported results of a comparison with Landrace pigs, Iberian pigs show about 20-30% greater rates of muscle protein synthesis, but lower relative and absolute weight of *biceps femoris*, *longissimus* and *semimembranosus* muscles than Landrace [57]. Under similar nutritional and physiological conditions, protein turnover as well as the protein synthesis to protein deposition ratio may differ between Iberian and leaner breeds, resulting in dissimilar protein deposition rates [58]. Our results of differential gene expression support this hypothesis and further specify the potential pathways and genes responsible of the metabolic differences observed.

In summary, the performed comparison of the muscle transcriptome of crossbred and purebred IB piglets allows to highlight several important biological functions narrowly related to their respective muscle characteristics. Muscle tissue development and ECM organization are strongly upregulated in DU×IB, in concordance with greater muscle and connective tissue development, characteristic of this genetic type. Genes and functions upregulated in IB are related to lipid and protein metabolism and are also in agreement with phenotypic traits. Higher lipid metabolism and protein catabolism are coherent with the higher fattening and lower protein deposition in IB muscles. These results provide potential mechanisms to explain the singular growth and fattening phenotype of Iberian pigs, consistent in an earlier adypocite differentiation and hypertrophy and lower protein deposition to synthesis ratio of their muscles. Time-course studies of the differential expression along growth would help to improve the understanding of the metabolic and development differences between genetic types here observed.

### Identification of transcriptional regulators potentially involved in the expression changes between genetic types

The magnitude of differential expression does not necessarily indicate biological significance [59]. A very small change in expression of a particular gene may have important physiological consequences if the protein encoded by this gene plays a regulatory role. Downstream genes usually amplify the signal produced by this regulator, thereby increasing their chance to be detected as DE by standard statistical methods. But the chance of a regulatory gene for being selected is small when focussing on the magnitude of differential expression. Regulatory Impact Factor (RIF) metrics have been developed to infer transcriptional regulation from gene expression data by identifying critical regulatory factors (RF) to explain the expression differences observed. RIF metrics are not dependent on the differential expression of the RF, increasing the biological knowledge that can be derived from gene expression experiments [60, 61]. RIF assigns extreme scores to those RF that are consistently most differentially co-expressed with highly abundant and highly DE genes (RIF1), or to those RF with the most altered ability to predict the abundance of DE genes (RIF2). We performed the prediction of RF with extreme RIF *z*-scores for the whole dataset of DE genes, and also for the genes included in each of the main gene networks detected with IPA.

#### a) Prediction of regulatory factors for the whole dataset of DE genes (256)

Regulatory factors potentially involved in the regulation of the metabolic processes differing in both genetic types were identified by their RIF1 and RIF2 *z*-scores. We performed the RIF study for 310 RF (309 TF and *MSTN* gene, see methods for description) included in the filtered array. We identified 29 RF with extreme *z*-scores for RIF1 and/or RIF2 parameters (Table 5). Sixteen RF showed extreme values for RIF1 and sixteen for RIF2. *KLF11* showed the most extreme score according to RIF1 (-3.79 SD units), and *ZHX2* had the most extreme score according to RIF2 (3.45 SD units). The genes *SIX4*, *EYA2* and *KLF11* were found to have extreme scores for both RIF1 and RIF2.

**Table 5:** **RIF prediction for regulatory factors in the whole dataset of DE genes**

***Probe set***	***Gene symbol***	***Gene***	***RIF1 z-***score^§^	***RIF2 z-***score^λ^
Ssc.10025.3.S1_at	*CEBPD*	CCAAT/enhancer binding protein (C/EBP). delta	-3.152	-0.017
Ssc.1012.1.S1_at	*ZNF277*	zinc finger protein 277	-1.251	2.751
Ssc.10128.1.A1_at	*SIX4*	SIX homeobox 4	-2.986	3.127
Ssc.13567.1.A1_at	*ZHX2*	zinc fingers and homeoboxes 2	-0.921	3.454
Ssc.14573.1.S1_at	*EYA2*	Eyes absent homolog 2	-2.907	3.048
Ssc.16976.1.S1_at	*SREBF2*	Sterol regulatory element binding transcription factor 2	-0.970	2.533
Ssc.19163.1.S1_at	*MXI1*	MAX interactor 1. dimerization protein	-1.860	3.443
Ssc.19313.1.A1_at	*MAX*	MYC associated factor X	-3.270	-1.671
Ssc.19537.1.S1_at	*IRF2*	Interferon regulatory factor 2	-2.701	-0.615
Ssc.2001.1.A1_at	*LHX6*	LIM homeobox 6	-1.383	-2.847
Ssc.21096.1.S1_at	*PAX2*	Paired box 2	-0.895	2.378
Ssc.22470.1.S1_at	*PER3*	Period circadian clock 3	-2.258	0.824
Ssc.23498.1.S1_s_at	*MSTN*	Myostatin	-1.930	2.582
Ssc.24606.1.A1_a_at	*PPARGC1B*	Peroxisome proliferator-activated receptor gamma coactivator 1 beta	-2.896	0.167
Ssc.26039.1.S1_at	*RORA*	RAR-related orphan receptor A	-3.597	1.736
Ssc.2719.1.A1_at	*MTA3*	Metastasis associated 1 family, member 3	-1.805	-2.555
Ssc.27410.1.S1_at	*MYCN*	v-myc avian myelocytomatosis viral oncogene neuroblastoma derived homolog	-2.823	-0.783
Ssc.27576.1.S1_at	*MN1*	Meningioma (disrupted in balanced translocation) 1	-2.263	-0.086
Ssc.27622.1.S1_at	*KLF11*	Kruppel-like factor 11	-3.789	2.701
Ssc.27964.2.S1_at	*GATA3*	GATA binding protein 3	0.063	-2.061
Ssc.29855.1.A1_at	*CDCA7*	Cell division cycle associated 7	-2.346	0.220
Ssc.30288.1.A1_at	*GRHL3*	Grainyhead-like 3 (Drosophila)	0.489	-2.038
Ssc.30799.1.A1_at	*DACH1*	Dachshund family transcription factor 1	0.772	-2.329
Ssc.3355.1.S1_at	*HDAC1*	Histone deacetylase 1	0.747	-1.971
Ssc.4212.1.A1_at	*ELK3*	ELK3, ETS-domain protein (SRF accessory protein 2)	-2.349	-1.032
Ssc.6697.1.S1_at	*SOX4*	SRY (sex determining region Y)-box 4	-3.143	-0.718
Ssc.8529.1.A1_at	*ZFP36L1*	ZFP36 ring finger protein-like 1	-3.282	1.299
Ssc.9136.1.S1_at	*CCRN4L*	CCR4 carbon catabolite repression 4-like (S. cerevisiae)	-1.056	-2.354
Ssc.9298.1.A1_at	*IRX3*	Iroquois homeobox 3	-2.538	-0.692

^§^Bootstrap 99% and 95% confidence intervals for *RIF1* z-scores: -3.288/2.754 and -2.155/1.898, respectively.

^λ^Bootstrap 99% and 95% confidence intervals for *RIF2* z-scores: -2.594/3.007 and -1.966/2.030, respectively.

RIF metrics allow the identification of relevant RF even not being DE. The critical RF identified in this study were not DE except *MAX* gene, which showed 1.4× upregulation in the IB group. As expected, RIF metrics identified RF which have been previously shown to have an important role in regulating myogenesis and adipogenesis, but also other ones with less known function. Myostatin (*MSTN*) is a member of the transforming growth factor beta (*TGF*β) superfamily that inhibits muscle differentiation and growth during myogenesis [62]. Its expression is negatively related with muscle mass. In fact, although Myostatin is not a transcription factor, it transmits the signal of TGF-beta cytokines activating multiple intracellular pathways resulting in phosphorylation and activation of downstream Smad proteins and other signalling molecules (e.g. Akt, MAPK, mTOR and Src). These molecules translocate to the nucleus, bind to DNA and regulate transcription of many genes by direct binding to the target gene promoter or through the interaction with transcriptional cofactors in a cell-type-specific manner [63, 64]. Although we do not detect its differential expression between genetic types, *MSTN* gene is predicted to be a main regulator of the transcriptome differences observed between them. This is in agreement with findings obtained in cattle by Hudson et al. [61, 65], which showed that *MSTN* is the RF with the highest evidence of contributing to differential expression in muscle in the absence of any demonstrable differential expression of the regulator itself. *GATA3* transcription factor is crucial in a variety of developmental processes including adipogenesis [66], and a negative regulator of TGFβ- and Smad4 signaling [67]. Five RF showing extreme RIF1 or RIF2 z-scores have been reported as key regulators of myogenesis, muscle cell differentiation and growth: *SIX4* and *EYA2* which show a joint and interactive role on activating key muscle determination genes [68, 69], *KLF11*[70], *SOX4*[71], and *HDAC1*[72]. Interestingly, three TFs known to be functionally related and involved in transcriptional regulation of cell proliferation are detected as potential regulators. These are the *MAX*, *MXI1* and *MYCN* genes. The MXI1 and MYCN proteins compete for interacting with MAX to form heterodimers, which compete for binding to target sites for transcriptional regulation. Whereas the MYCN-MAX complexes induce transcriptional activation, the MXI1-MAX heterodimers repress transcription [73]. Thus, the balance among the different TFs determines the proliferation and tissue growth rate. Also, the *CDCA7* gene codes for another TF which regulates cell proliferation being a target of MYC-dependent transcriptional regulation [74]. The GRHL3 transcription factor is involved in development and migration of endothelial cells being considered an angiogenic factor [75], and participates in the regulation of actin cytoskeleton organization. Interestingly, the *Iroquois Homeobox 3* gene (*IRX3*) is identified in our work as a potential regulator for the gene expression differences observed between genetic types. This gene has very recently been proposed as a novel determinant of body mass and composition [76].

Also, RF specifically related to adipogenesis and lipid metabolism were predicted to regulate the expression changes. Peroxisome Proliferator-Activated Receptor Gamma Coactivator 1 β (*PPARGC1B*) is involved in fat oxidation, non-oxidative glucose metabolism, mitochondrial biogenesis, and the regulation of energy expenditure [77]. It stimulates lipogenic gene expression by activating *SREBP* transcription factor family. Consistently, *SREBF2*, a known gene with role on cholesterol homeostasis and control of lipid levels, is also predicted to regulate the set of DE genes. CCAAT/enhancer binding protein delta (*CEBPD*) belongs to the *CEBP* family of adipogenic TF and plays a crucial role in mitotic clonal expansion in the early stages of adipocyte differentiation [78]. Retinoic acid receptor related orphan receptor alpha (*ROR*α) is an orphan member of the nuclear receptor superfamily of TF related to lipid homeostasis. It influences genes associated with lipid and carbohydrate metabolism, fatty acid oxidation, insulin signalling, LXR nuclear receptor signalling, and Akt and AMPK signalling in mouse skeletal muscle [79]. Also it influences the expression of *SREBP* and *PPARGC1B* TF. Hence, these RF with extreme RIF z-scores are important metabolic regulators which could be especially relevant for the differences in adipocyte differentiation pattern, lipid metabolism, energy balance regulation and fat deposition between muscles of both genetic types.

The identification of different RF corresponding to the same pathways (*MAX-MXI-MYCN-CDCA7, MSTN-GATA3, EYA2-SIX4, PPARGC1B-CEBPD-GATA3-SREBF2*) is an indication of the reliability of the results and the involvement of the whole pathways in the expression differences found between the compared genetic types.

The RIF approach also gives novel findings regarding functional relationships not previously reported in muscle. Not much is known about *ZHX2*, *LHX6*, *PAX2, PER3*, *IRF2*, *ZNF27, ELK3* or *ZFP36L1* regulatory factors in relation to muscle structure or function, although some of them have known roles on developmental processes in other tissues. Also, other ones show key roles in the regulation of cell proliferation and differentiation and are associated with tumour development as *MTA3, MN1* or *DACH1*.

In order to reinforce the reliability of the RIF study, the potential compatibility of the RIF-predicted RF and DE genes was also explored at the DNA sequence level, to analyze the presence of transcription factor binding sites (TFBS) in the DE genes promoters. This study was applied to the DNA-binding and sequence-specific transcription factors, which are the ones analyzed by the Genomatix software. On average, we detected TFBS for the RIF-predicted DNA-binding TF in a 60% of the DE genes promoters. This software also allowed the identification of TF for which the number of TFBS in the DE genes was significantly enriched. The transcription factors *MXI1*, *MAX*, *MYCN, ELK3, GRHL3, SIX4, PAX2, SREBF2* and *KLF11* showed significantly higher number of matches with the promoter sequences than the value expected by chance (Benjamini-corrected *P* values =6×10^-16^ for *MXI1*, *MAX* and *MYCN*; 0.001 for *ELK3*, 0.0008 for *GRHL3*, 0.037 for *SIX4*, 3.5×10^-8^ for *PAX2*, 0.0008 for *SREBF2* and 5.7×10^-25^ for *KLF11*).

#### b) Prediction of regulatory factors for the main networks of DE genes detected with IPA

Several gene networks detected by IPA were selected because of its potential role on the phenotypic differences between genetic types, and RIF metrics were also calculated for the DE genes included in each one of these three main networks.

For the first NW (Development, growth, proliferation), we detected 31 potential regulators (Additional file 3). Out of them, sixteen were coincident with RF previously identified in the whole dataset study and fifteen RF were new ones. Among these, several RF with known roles in the functions affected by this NW can be identified, as *MYF6* which is a main regulator of muscle cell differentiation [80], *KLF10* which inhibits growth, acting as negative regulator of cellular proliferation [81], or *ETS2*, which regulates genes related with development and adipogenesis [82]. Moreover, some of the network-specific identified regulators have a role in cell differentiation or proliferation (*KLF5*, *MAFF* or *SRF*).

The relationships found among RF and DE genes were separately analyzed for each NW employing IPA and GENEMANIA [83] tools. Both softwares allow finding relationships in a set of input genes, using a very large set of functional association data, including protein and genetic interactions, pathways, co-expression, co-localization and protein domain similarity. For NW1, all the RF detected with RIF algorithm were predicted to be biologically related with the molecules included in the NW, except *LHX6*. According to GENEMANIA each RF was found to be related to a mean of nine molecules in the NW. The genes *CEBPD*, *ZHX2* and *RORA* were the most connected ones.

For the genes included in the second NW (cellular assembly), 27 RF were detected with extreme RIF scores (Additional file 3). Most of them (21) coincide with previously identified ones. Among the 6 new ones, *FOSB* gene may be highlighted as a regulator of cell proliferation, differentiation and transformation, known to regulate cell-matrix adhesion [84]. As for the first NW, most relationships between RF and DE genes were confirmed to have a biological support. This NW showed and average number of eight relationships among RFs and DE genes, and several predicted RF were connected to many DE genes, suggesting a more important regulatory role (*FOSB, CEBPD, SOX4, RORA, ZHX2, ZFP36L1* and specially the complex *MXI-MAX-MYC*).

For the third NW (proteolysis), which includes only genes upregulated in IB, we detected 29 RF. Out of them, 12 genes were common with the global study and a few more were detected previously in the other networks, but 13 RF were specific for this NW (Additional file 3). Among these, we can highlight some interesting genes, as *ETV5*, potentially related to energy balance [85], and the *HOXA7*, *HOXA9*, *HOXB7* and *KLF4* genes involved in cellular proliferation and differentiation, cell fate determination and adipogenesis [86–88]. On the other hand, the identification of *MSTN* and *STAT5B* as regulators of this NW is interesting, as GH effects on muscle growth are known to act via STAT5B, which regulates the abundance of mature myostatin by proteolytic cleavage [89]. Also, several TF related to lipid metabolism are identified (*RXRB, PER2*). For NW3 (proteolysis), all the detected RF were predicted to be significantly related with DE genes in this NW. However, most of them showed scarce known connections with the DE genes, with a mean number of five connections. Only the *KLF4, ZHX2* and *EYA2* genes were related to a higher number of DE genes according to the available information.

The potential relationship of the RIF-predicted RF and DE genes was also explored at the DNA sequence level using the Genomatix software. In each NW, we detected TFBS for the RIF-predicted DNA-binding transcription factors in a mean of 58% of the DE genes’ promoters (54, 58 and 63% for networks 1, 2 and 3, respectively). The results allowed the identification of TF for which the number of TFBS in the DE genes was significantly higher than the number expected by chance. For NW1, the transcription factors *MXI1*, *KLF5*, *KLF10*, *KLF11* and *SREBF2* showed significantly higher number of matches than expected (Benjamini-corrected *P* values =0.003 for the first four genes and 0.005 for *SREBF2*). Transcription factor binding sites for *KLF11* were significantly overrepresented in the set of DE genes included in NW2 (Benjamini-corrected *P* value =0.03). At last, for NW3 the transcription factors *MXI1*, *MAX*, *KLF4 and KLF11* were highlighted (Benjamini-corrected *P* values =0.006, 0.006, 0.03 and 0.03, respectively). Thus, although this tool is only applicable to sequence-specific DNA-binding TF, these results reinforce the previous evidences, highlighting the transcription factors *MXI1*, *MAX* and *KLFs* as the most solid findings according all the available information (RIF z-scores, biological relationship and sequence information).

On the other hand, among the RF predicted in the global study of DE genes and in each functional NW, we found RF which consistently appear with elevated RIF z-scores in all analyses (*MSTN, MXI1, SIX4, EYA2, IRX3* and *ZHX2*), which may be responsible of a large part of the gene expression differences detected in muscles from crossbred and purebred Iberian piglets. A regulatory role of these RF on the main phenotypic differences between the compared genetic types can be thus suggested. Also, RF with potential role in specific functions can be identified, as the RF detected exclusively for the proteolysis network, most of which have poorly known roles.

#### c) Differential correlation among RF and DE genes in the two genetic types

The correlation structure of gene expression conveys important biological information far beyond the marginal measures of differential expression. In fact, co-expression measures can be used to uncover significant features of cellular control and may help in the determination of gene function [59]. RIF’s scores measure differential co-expression (differential connectivity) between two conditions, which integrates three sources of information: the amount of differential expression of DE genes; the abundance of DE genes and the change in correlation existing between the RF and the DE genes [60, 61]. In order to better understand the relationships of the RF predicted with the DE genes, we studied the correlations among their expression values in each one of the genetic types. These correlations were calculated for the three functional networks (Additional file 4). For the first NW, gene expression correlations were calculated between 31 RF and 29 DE genes (899 correlations in each genetic type). Among these, 28 significantly differed between genetic types (FDR <0.10). For the second NW, we calculated in each genetic type the 594 correlations between 27 RF and 22 DE genes, with 80 resulting statistically different between genetic types (FDR < 0.10). Finally, for the third NW, 29 RF and 20 DE genes were employed for the correlation study (580 correlations for each group). In this case 33 correlations were significantly different (FDR < 0.10). For NW 2, those 80 correlations significantly different between genetic types were represented employing Cytoscape 2.8.0 [90] (Additional file 5).

For the first NW, a higher number of correlations significantly differing from zero were observed in IB piglets’ data (Additional file 4). This would suggest a higher activity of transcriptional regulation in purebred animals (either induction or repression), in relation to the biological functions affected by this NW. This would be in agreement with the enriched biological function of transcriptional regulation observed in the IB group in the GO study with DAVID tool (Table 3 and additional file 2). For NW2, a higher proportion of negative correlations was observed in IB dataset (Additional file 5). Several RF involved in the regulation of muscle growth, as *MSTN, RORA, SIX4, KLF11* or *EYA2*, are negatively correlated with the DE genes (which are mostly upregulated in crossbred animals in this NW) in IB dataset, but the correlations are not significantly different from zero in DU×IB piglets. This suggests a transcriptional repression in IB animals of genes involved in ECM development and function. In DU×IB piglets, some RF correlate with the DE genes (*LHX6, MAX, MTA3*), with most correlations being positive. In NW3, the differential correlation allows to highlight several RF which show opposite behavior in both genetic types. The genes *ZHX2* and *EYA2* are correlated with several of the DE genes in IB but not in DU×IB, while *LHX6* and *MAX* show the opposite results. These RF may have a role in the expression differences observed between genetic types regarding proteolytic genes.

## Conclusions

In this study, purebred and crossbred Iberian piglets differing in muscle traits were compared at the transcriptome level, and a remarkable number of DE probes were detected. The study of DE genes allowed us the identification of biological functions and pathways with relevant role in the differences in development of muscle and intramuscular adipose tissues between the studied genetic types. Muscle development and ECM components are clearly upregulated in crossbred piglets, and the results indicate its main role in the differentiation and development of muscular, adipose and connective tissues, and thus on many growth and meat quality parameters. Over-expression of lipid metabolism genes in purebred Iberian muscle agrees with an earlier adipocyte development in purebred Iberian pigs. In addition, proteolysis pathways were upregulated in purebred Iberian muscle, with potential negative consequences on protein deposition and lean growth.

Apart from measuring differential expression across genetic types, we studied differential co-expression with regulatory factors, thus improving the understanding of the gene expression data and increasing the biological knowledge generated from the experiment. Several RF were identified which could be responsible of the transcriptional regulation of muscles of both genetic types. Some of them have known roles on myogenesis and cell proliferation (as *MSTN*, *MYF6*, *SIX4*, *EYA2, MXI1, MAX, MYCN*, *KLF11, IRX3*) or adipogenesis (as *PPARGC1, SREBF2* or *CEBPD*). Also transcriptional regulators for the specific affected functions were identified, which deserve further attention, as *ETS2*, *FOSB*, *KLF4* or *PAX2*. At last, RF prediction and correlation study suggest a transcriptional repression of genes involved in muscle growth and ECM function in muscles of purebred piglets.

Results provide candidate genes (DE genes and putative regulatory factors) to explain the phenotypic differences that characterize the genetic types compared. The identification of polymorphisms responsible for these expression changes would be the following step for the practical application of these findings to improve meat quality.

## Methods

### Animal material

The current study was carried out at the facilities of the CIA Deheson del Encinar (Toledo, Spain). Animal manipulations were performed in compliance with the regulations of the Spanish Policy for Animal Protection RD1201/05, which meets the European Union Directive 86/609 about the protection of animals used in experimentation. The experiment was specifically assessed and approved (report CEEA 2010/003) by the Spanish National Institute for Agricultural and Food Research and Technology (INIA) Committee of Ethics in Animal Research. Two groups of Iberian sows were either mated with Iberian boars or inseminated with Duroc semen. Thirteen contemporary litters were generated (ten of purebred and three of crossbred animals). At weaning (28 days) at least one male piglet was randomly chosen from each litter. A total number of 14 piglets of each genetic type were slaughtered and eviscerated. Loin tissue samples were collected from the carcasses at the level of the last rib and stored at -80°C. Samples were employed for composition and gene expression studies.

### Tissue composition analyses

Intramuscular fat was obtained as proposed by Marmer and Maxwell [91]. Longissimus dorsi muscle fat extracts were methylated in the presence of sulphuric acid and analysed by gas chromatography. Fatty acid methyl esters (FAMEs) were identified by gas chromatography as described elsewhere [92] using a Hewlett Packard HP-6890 (Avondale, PA, USA) gas chromatograph equipped with an automatic injector, a flame ionization detector and a capillary column (HP-Innowax, 30 m × 0.32 mm i.d. and 0.25 μm polyethylene glycol-film thickness) (Agilent Technologies Gmbh, Germany). A split ratio of 1:50 and a temperature program of 170 to 245°C were used. The injector and detector were maintained at 250°C. The carrier gas (helium) flow rate was 2 ml/min. Results were expressed as grams per 100 grams of detected FAMEs.

### Microarray study

#### RNA isolation and microarray hybridization and analysis

Loin muscle RNA from 28 animals (14 of each genetic type), was isolated using RiboPure RNA isolation kit (Ambion) following the manufacturer’s recommendations. RNA obtained was quantified using a NanoDrop equipment (NanoDrop Technologies, Wilmington, USA) and RNA quality was assessed with an Agilent bioanalyzer device (Agilent Technologies, Palo Alto, USA). The RNA Integrity Number (RIN) values obtained showed an average of 8.5 ± 0.4, thus assuring their homogeneity and high quality. A non-competitive hybridization with the GeneChip^®^ Porcine Genome Array (Affymetrix, Santa Clara, CA, USA) was performed in two successive series of 16 and 12 samples. This microarray contains 24,123 probe sets that interrogate around 23,250 transcripts from 20,201 Sus scrofa genes. The RNA samples were transferred to the Institut de Recerca Hospital Universitari Vall d’Hebron (Barcelona, Spain) for reverse transcription, fluorescent labeling, hybridization on chips and scanning. Briefly, for each sample 5 μg of total RNA was reverse-transcribed into cDNA molecules, labeled with biotin and hybridized to the high density oligonucleotide chip. Hybridizations were done according to Affymetrix standard protocols and expression data were generated with GeneChip Operating Software (GCOS). All protocols followed the MIAME recommendations [93] developed by the Microarray Gene expression Database Group (http://www.fged.org/). The data sets supporting the results and discussed in this publication have been deposited in NCBI’s Gene Expression Omnibus repository [94] and are accessible through GEO Series accession number GSE53029 (http://www.ncbi.nlm.nih.gov/geo/query/acc.cgi?acc=GSE53029).

#### Quality control, normalization and filtering of expression data

Microarray data quality evaluation was carried out with the “Affy” and “Sympleaffy” packages of Bioconductor software (http://www.bioconductor.org/) [95]. All the 28 hybridizations performed overcame the quality control and were used for statistical analysis. Normalization was conducted to reduce technical variation between chips. GCRMA normalization was carried out with BRB-Array Tools (v. 3.7.1) (http://linus.nci.nih.gov/BRB-ArrayTools.html) [96]. A filtering step was performed to exclude from the analyses the genes showing minimal variation across the set of arrays. This filtering has been shown to improve the power to detect differential expression [97]. Only genes displaying more than 20% of expression values over ± 1.5 times the median expression of all arrays were used for further analysis. From the total 24,123 probe sets of the array, 5,226 spots overcame these filtering conditions and were used in the statistical analysis of differential expression.

#### Statistical analysis of microarray data

Normalized microarray expression data (background corrected and base-2 logarithmic-transformed) were analyzed through Bayesian inference using the GEAMM v.1.6 software [98]. The following model was used for searching the effects on expression data of both genetic types:

where *y* (*pq* × 1 elements) is the vector of gene expression data sorted by successfully hybridized array (*q* = 28) and probe within array (*p* = 5,226), and influenced by the overall effect of each array (a) as well as discrete (*Di*) within-probe effects (genetic type and hybridization series), both with dimensions 1 × *p*. All the unknowns in the model were sampled from their joint posterior distribution by Gibbs sampling [99]. Additional details of the performed Bayesian procedure are reported by Casellas et al. [98].

Inferences were made on the probe-specific difference between *Di* levels from the appropriate posterior distributions summarized by its mean, standard deviation and posterior probability (*PP*) above (negative mean) or below (positive mean) zero. When the magnitude of *PP* values is very small, it provides substantial evidence on the differential expression of a given probe. In this study these posterior probabilities were treated as *P*-values for calculating their maximum value under multiple testing within the false discovery rate (FDR) approach of Benjamini and Hochberg [100]. Because the probe expression values are correlated, the effective number of independent probes (*M*_eff_ = 4,068) was calculated according to Moskvina and Schmidt [101] and used in the calculation of FDR value.

#### GeneChip porcine genome array reannotation

Probes were annotated using the latest Affymetrix annotation file available (http://www.affymetrix.com/catalog/131488/AFFY/Porcine-Genome-Array#1_1). Owing to the possibility of missannotation, gene annotation of the DE genes was confirmed from the available sequence used by Affymetrix to design the probes in the array. Each sequence was analyzed by BLAST [102] to confirm the gene annotation, based on homology with other genomes such as human, mouse or bovine, among others.

#### Gene ontology and functional annotation

To study the functionality of the DE genes we used Gene Ontology (GO) information. The biological interpretation of the data was carried out using the DAVID 2008 database tool [29] which provides batch enrichment analyses to highlight the most relevant GO terms associated to a gene list. This tool detects overrepresented functional gene categories in the gene list compared with a background genome, which in our case was the set of genes present on the filtered array. Significance levels are calculated following a modification of Fisher’s exact test (also named EASE score). Functional terms with *P*-values lower than 0.05 are usually considered enriched in the annotation categories. A multiple testing-corrected *P*-value was also calculated using Benjamini and Hochberg algorithm [100], and GO terms with Benjamini-corrected *P* < 0.10 were considered significant (Additional file 2). Functional annotation clustering was also performed by employing biological process GO terms. Usually, an enrichment score of 1.3, which is equivalent to non-log scale *P*-value of 0.05, is employed as threshold for cluster significance [29]. Further, we calculated the geometric mean of the Benjamini-corrected *P* values of the GO terms included in each functional cluster, and retained those clusters with values lower than 0.10 (Tables 3 and 4).

As a complementary approach, Ingenuity Pathway Analysis, Ingenuity Systems (http://www.ingenuity.com) bioinformatics tools were employed to identify and characterize biological functions, gene networks and canonical pathways affected by the treatment. These tools integrate the Ingenuity Knowledge Base of gene-to-gene or protein-to-protein interaction information and the annotated data from the gene expression experiment to generate biological relevant gene regulatory networks. Networks are collections of interconnected molecules assembled by a NW algorithm. Each connection represents known relationships between the molecules, found in the Ingenuity Knowledge Base. Networks are created from “seed” molecules. IPA searches the Ingenuity Knowledge Base for molecules that are known to biologically interact with the seed molecules and makes connections based on the findings. The most highly connected molecules in the DE list and the knowledge base are consolidated into Networks. Network analysis returns a score that ranks networks according to their degree of connectivity and relevance to the network eligible molecules in the dataset [103]. The network score is based on the hypergeometric distribution and is calculated with the right-tailed Fisher’s exact test. The score is the negative log of this *P*-value. Molecules that demonstrate direct and indirect relationships to other genes, or proteins were integrated into the analysis. The IPA Canonical Pathways Analysis identified the pathways from the Ingenuity Pathways Analysis library of canonical pathways that were most significant in our dataset. The significance of the association between the dataset and the canonical pathway was measured with Fischer’s exact test, to calculate a *P*‒value determining the probability that the association between the genes in the dataset and the canonical pathway is explained by chance alone.

The global list of 256 DE genes affected by genetic type and the two partial lists of 102 and 154 genes upregulated in IB and DU×IB, respectively, were explored using both the DAVID tool and the core analysis function included in IPA Analysis.

### Search of regulatory factors with RIF metrics

RIF1 and RIF2 metrics [60, 61] were calculated for the whole set of DE genes obtained conditional on genetic type (256 genes), and also for the genes included in the most interesting networks identified by IPA software. A manually curated census of 1072 regulatory factors was obtained from Perez-Montarelo et al. [104]. This list was elaborated from previous publications [105, 106] and transcription factor databases (http://www.bioguo.org/AnimalTFDB/; http://www.hprd.org/). Out of those 1072 RF, 310 had probes present in the filtered Affymetrix array, and were used for the RIF analysis (Additional file 6). This list includes sequence-specific DNA-binding transcription factors, but also other transcription factors and cofactors. The *MSTN* gene was included in the list despite not being a transcription factor, due to its relevant and widely known involvement in muscle growth regulation in mammals. The RIF1 and RIF2 values were computed for the *i*^th^ RF as follows:



and

where *n*_*de*_ is de number of DE genes,  and  the estimated average expression and differential expression of the *j*^*th*^ DE gene, *r*1_*ij*_ and *r*2_*ij*_ the co-expression correlation between the *i*^*th*^ RF and the *j*^*th*^ DE gene in each one of the genetic types and being *e*1_*j*_ and *e*2_*j*_ the expression of the *j*^*th*^ gene in each genetic type [59]. Both RIF measures for each analyzed RF were transformed to standardized *z*-scores by substracting the mean and dividing by its standard deviation. We identified relevant RF as those with extreme RIF z-scores according to the corresponding confidence intervals (CI) calculated by bootstrap. In each iteration of bootstrapping, a set of n_de_ = 256 genes (n_de_ = 29, 22 and 20 genes for the networks 1, 2 and 3) were randomly selected from the 5,226 probes of the filtered array, and the RIF1 and RIF2 z-scores of the 310 RF were calculated. The procedure was repeated 10,000 times for each scenario to obtain the corresponding 95 and 99% CI intervals of both z-scores.

Different softwares were employed to analyze the compatibility of RIF-predicted RF with the DE genes. IPA (IPA, http://www.ingenuity.com) and Genemania (http://genemania.org) [83] softwares were employed to check for available information about biological relationships between RF and DE genes, and for the construction of networks joining both types of molecules. Genomatix software suite (http://www.genomatix.de/) was employed for the verification of the presence of TFBS in the promoter sequences of the DE genes, which can bind the transcription factors predicted with RIF metrics. The *Gene2promoter* tool was employed to retrieve promoter sequences for the DE genes from *Eldorado* database. The selected promoters were subjected to the search of TFBS with the *CommonTFs* tool, which searches multiple sequences for common TFBSs. For this search, user-defined matrix subsets were created including the matrix families corresponding to the set of RF identified in each step (whole study with the 256 DE genes and each one of the networks), obtained from *MatBase*. Matrix families were available for a subset of the RIF-predicted RF (Additional file 6). The *CommonTFs* tool returns the number of sequence matches found between transcription factors and gene promoters included in the analysis and also a *P* value, which denotes the probability to obtain an equal or greater number of sequences with a match in a randomly drawn sample of the same size as the input sequence set. A multiple testing-corrected *P*-value was also calculated using Benjamini and Hochberg algorithm [100].

Pearson correlations between the gene expression values of RIF-predicted RF and DE genes in each one of the three IPA networks were calculated. The correlations were calculated for the whole set of available animals, and also separately for IB and DU×IB animals. Statistically significant differences in the correlations between both genetic types were identified for each network (Benjamini-corrected *P* values < 0.10). Because the probe expression values are correlated, the effective numbers of independent RF and DE genes were calculated according to Moskvina and Schmidt [101] and used in the calculation of Benjamini-corrected *P* values. Correlations which differ between genetic types were graphically represented employing Cytoscape 2.8.0 [90].

### Validation of DE by quantitative PCR (qPCR)

The RNA obtained from loin of the 28 animals under study was employed to perform the technical validation of the differential expression of some probes and also to assess the expression of one candidate gene absent in the microarray data (*DLK1*). First-strand cDNA synthesis was carried out with Superscript II (Invitrogen, Life Technologies, Paisley, UK) and random hexamers in a total volume of 20 μl containing 1 μg of total RNA and following the supplier’s instructions.

The expression of 19 genes (eight upregulated in DU×IB, eight upregulated in IB, two non-changed ones and one absent in the Affymetrix microarray) was quantified by qPCR. Primer pairs used for quantification were designed using Primer Select software (DNASTAR, Wisconsin, USA) from the available GENBANK and/or ENSEMBL sequences, covering different exons in order to assure the amplification of the cDNA. Sequence of primers and amplicon lengths are indicated in Table 6. Standard PCRs on cDNA were carried out to verify amplicon sizes. Transcript quantification was performed using SYBR Green mix (Roche, Basel, Switzerland) in a LightCycler480 (Roche, Basel, Switzerland). The qPCR reactions were prepared in a total volume of 20 μl containing 2.5 μl of cDNA (1/20 dilution), 10 μl of SYBR Green mix (2X) and 0.15 μM of both forward and reverse primers. As negative controls, mixes without cDNA were used. Cycling conditions were 95°C for 10 min, followed by 45 cycles of 95°C (15 s) and 60°C (1 min) where the fluorescence was acquired. Finally, a dissociation curve to test PCR specificity was generated by one cycle at 95°C (15 s) followed by 60°C (20s) and ramp up to 95°C with acquired fluorescence during the ramp to 0.01°C/s. Data were analysed with LyghtCycler480 SW1.5 software (Roche, Basel, Switzerland). All points and samples were run in triplets as technical replicates and dissociation curves were carried out for each individual replicate. Single peaks in the dissociation curves confirmed the specific amplification of the genes. For each gene, PCR efficiency was estimated by standard curve calculation using four points of cDNA serial dilutions. Values of PCR efficiency are indicated in Table 6. Mean Cp values were employed for the statistical analyses of differential expression. Data normalization was carried out using the two most stable endogenous genes selected out of: *GAPDH*, *B2M*, *TBP* and *ACTB*. Endogenous genes stability measures were calculated from Genorm software [107]. The *GAPDH* and *ACTB* genes were finally employed.

**Table 6:** **Information on the primer pairs used for quantitative real-time PCR analysis**

Gene symbol	Gene name	Genbank Acc. number	Primer sequences 5′-3′	Amplicon size (bp)	***Efficiency (%)***
*IGF2*	Insulin-like growth factor 2	NM213883	GCCGCTGCTCGTGCTGCTCGTCTT	151	86
			GCTTGCCGGCCTGCTGAA		
*KERA*	Keratocan	XM001927128	GTGGCCTTCCTGAGACTAAACC	198	89
			AGGGCATATCACAGAGACATTCAC		
*FMOD*	Fibromodullin	XM003130105	GCTGCTATATGTGCGGCTGTC	194	93
			AGAAACTGCTAATGGAGAACT		
*COL1A1*	Collagen alpha-1	EF136662	AGCCCAGCGTGCCCCAGAAGAA	164	88
			ACATCAGGCGCAGGAAGGTCAGC		
*FBN2*	Fibrillin 2	XM003123897	GGACGCTGCATACCTACTGT	201	96
			AATGCATGCTTGCTTGGTAGG		
*AEBP1*	AE binding protein 1	XM003134886	CGGCGGCATGGGCATCGTCAAC	233	90
			TGCCCTGCTCGTCCGTCACTACCC		
*LOX*	Lysyl oxidase	NM001206403	CTGAGATGCGCTGCGGAGGAAAAC	223	88
			TGGCATCAAGCAGGTCGTAGTGG		
*FKBP14*	FK506 binding protein 14	XM005673279	TTCCGGAACTTCTTTCCTGCTCT	250	91
			GGCTGACCATTGTTATGTTTGTGA		
*PSMD11*	Proteasome 26S subunit, non-ATPase	XM003131741	TCTTACGCCAGGCTTTGGAG	219	91
			CTGTGGTTCGAGCAGAGGTT		
*ALOX5AP*	Arachidonate 5-lipoxygenase-activating protein	NM001164001	TGGACTGATGTACCTGTTTGTGAG	213	94
			AGAGGGGAGACGGTGGTGGTGA		
*CASP4*	Caspase 4	XM003129812	AATATGCTTGGCGCTGTCAC	190	97
			TGGTGCTTCTCGAAGTTGGT		
*ELOVL6*	Fatty acid elongase 6	XR305072	AGAACACGTAGCGACTCCGAAGAT	182	96
			GACATGCCGACCGCCAAAGATAA		
*NFKBIZ*	NF-kappa-B inhibitor zeta-like	XM003132694	TATGATGGCCTGACTCCTCTACAC	196	91
			TGCGGCCACTTTTACGAT		
*ME1*	Malic Enzyme	XM001924333	TTTCCTGGAGTTGCCCTTGGTGT	213	90
			GGTGGCTGTCTTTTCTTGGTATGC		
*PLA1A*	Phospholipase A1 member A	XM003483312	TGTGGGCAGCTAGTGGAAGAAAGT	215	91
			TCCACGGCTGAAAAGTAGACACC		
*PON3*	Paraoxonase 3	HQ542303	ACGGGAGATATTTGGGCAGG	142	92
			TGTTGGCATACTCGGTGCTT		
*SCD*	Stearoyl-CoA desaturase	JN613287	TCCCGACGTGGCTTTTTCTTCTC	205	90
			CTTCACCCCAGCAATACCAG		
*ELOVL5*	Fatty acid elongase 5	ENSSSCG00000024149	CTTGCCGGGGGATTTTGGTTG	223	82
			TTGCGCAGGATGAAGAAGAAGGTG		
*DLK1*	Delta-like 1 homolog	NM_001048187	CGGGCCCTGCGTGATGAATGG	208	83
			AGGGCAGCGGCAGCGGAAGTC		

### Statistical analyses of tissue composition and qPCR gene expression data

The influence of genetic type on FA composition was separately analyzed for each fatty acid with a linear model fitting as systematic effects the mean and genetic type, and as random the full-sib family and residual effects. For the analysis of qPCR expression data Cp values were transformed to relative quantities using the comparative Cp method. This method is a relative quantification method in which relative gene expression quantities are calculated from Ct/Cp values by employing the specific PCR efficiency values previously calculated for each gene and making the values relative to the sample with higher expression, which is employed as calibrator (Qty = 10^-ΔCp/slope^) [108]. The qPCR expression data normalization was performed using normalization factors calculated with Genorm software (http://medgen.ugent.be/~jvdesomp/genorm/) from the *GAPDH* and the *ACTB* expression values. Relative quantities were divided by the normalization factors which were the geometric means of the two reference genes quantities. Normalized gene expression relative measures obtained were analyzed conditional on genetic type with a *t*-test. All the analyses were performed using the GLM procedure of SAS 9.1 (SAS Institute Inc., Cary, NC, USA).

The concordance correlation coefficient (CCC) between the fold-change values estimated in loin from microarrays and qPCR expression measures for the 18 genes was calculated to validate the global microarrays results [28].

### Availability of supporting data

The data sets supporting the results and discussed in this publication have been deposited in NCBI’s Gene Expression Omnibus repository [94] and are accessible through GEO accession number GSE53029 (http://www.ncbi.nlm.nih.gov/geo/query/acc.cgi?acc=GSE53029).

## Electronic supplementary material

Additional file 1: **Annotated list of probes showing differential expression between genetic types.** List and annotation of the 271 probes differentially expressed between l.dorsi muscle of purebred Iberian piglets and crossbred with Duroc (FDR < 0.10), at 28 d of age. Posterior probabilities (*PP*) for the genetic type effect and expression ratios (calculated from mean log_2_ intensities) are shown. (XLSX 35 KB)

Additional file 2: **Gene ontology terms.** List of significantly overrepresented GO terms (distributed over the three main categories) and KEGG pathways in the three analysed gene lists: the 256 DE genes between genetic types, the 154 genes upregulated in DUxIB and the 102 genes upregulated in IB. Parametric *P*-values are indicated along with Benjamini multiple test corrections (FDR < 0.10). (XLSX 41 KB)

Additional file 3: **Prediction of RF for the three functional networks.** RIF metrics were employed for the identification of RF potentially involved in the three functional gene networks detected with IPA software. Annotation and RIF z-scores are provided for the RFs identified in each network. Bootstrap 99% and 95% confidence intervals for RIF1 and RIF2 z-scores are indicated for each network, as inserted comments in the corresponding columns. (XLSX 28 KB)

Additional file 4: **Gene expression correlations between RF and DE genes.** Correlations were calculated among the expression values of RF and DE genes, for the three functional IPA networks, separately for the animals of each genetic type. For each network, magnitude and significance of the correlation in each genetic type and significance of the difference in the correlation between genetic types are shown (nominal *P*-values). Shaded lines are correlations significantly different in both genetic types (FDR < 0.10). (XLSX 229 KB)

Additional file 5: **Graphical representation of the correlations RF-DE genes in network 2 (Connective Tissue Disorders, Dermatological Diseases and Conditions, Cellular Assembly and Organization).** Gene expression correlations between RIF-predicted regulatory factors and DE genes, which were significantly different in both genetic types, were graphically represented with Cytoscape 2.8.0, for each genetic type. Light circle nodes represent regulatory factors. Squared nodes are DE genes, red ones are upregulated in IB while green ones are upregulated in DUxIB. Solid lines are significant correlations and dotted lines are non-significantly different from cero (in each genetic type). Blue lines are positive correlations and pink ones are negative correlations. (DOCX 785 KB)

Additional file 6: **Regulatory factors employed for the RIF analysis.** Annotated list of 310 regulatory factors with probes available in the filtered array and employed for the RIF analysis. The list includes sequence-specific DNA-binding transcription factors, other transcription factors and cofactors. Also *MSTN* is included,despite not being a TF, because of its known involvement in muscle growth regulation. Gene ontology and Genomatix annotation information is provided. (XLSX 469 KB)

## Abbreviations

CCC: Concordance correlation coefficient
CI: Confidence interval
DAVID: Database for annotation, visualization and integrated discovery
DE: Differentially expressed
DUxIB: Duroc x Iberian
FDR: False discovery rate
GEO: Gene expression omnibus
IB: Iberian
IMF: Intramuscular fat
ECM: Extracellular matrix
FA: Fatty acid
FAME: Fatty acid methyl esters
GO: Gene ontology
IPA: Ingenuity pathway analysis
MUFA: Monounsaturated fatty acid
NW: Network
*PP*: Posterior probability
qPCR: Real-time quantitative PCR
RF: Regulatory factor
RIF: Regulatory Impact Factor
TF: Transcription factor
TFBS: Transcription factor binding site.
