# Supplementary figures and images for: Longissimus dorsi transcriptome analysis of purebred and crossbred Iberian pigs differing in muscle characteristics
Source: BMC Genomics. 2014 May 31;15:413. doi: 10.1186/1471-2164-15-413 (PMC4070551; doi:10.1186/1471-2164-15-413)

**Network 2: ECM**

**IB DUxIB**


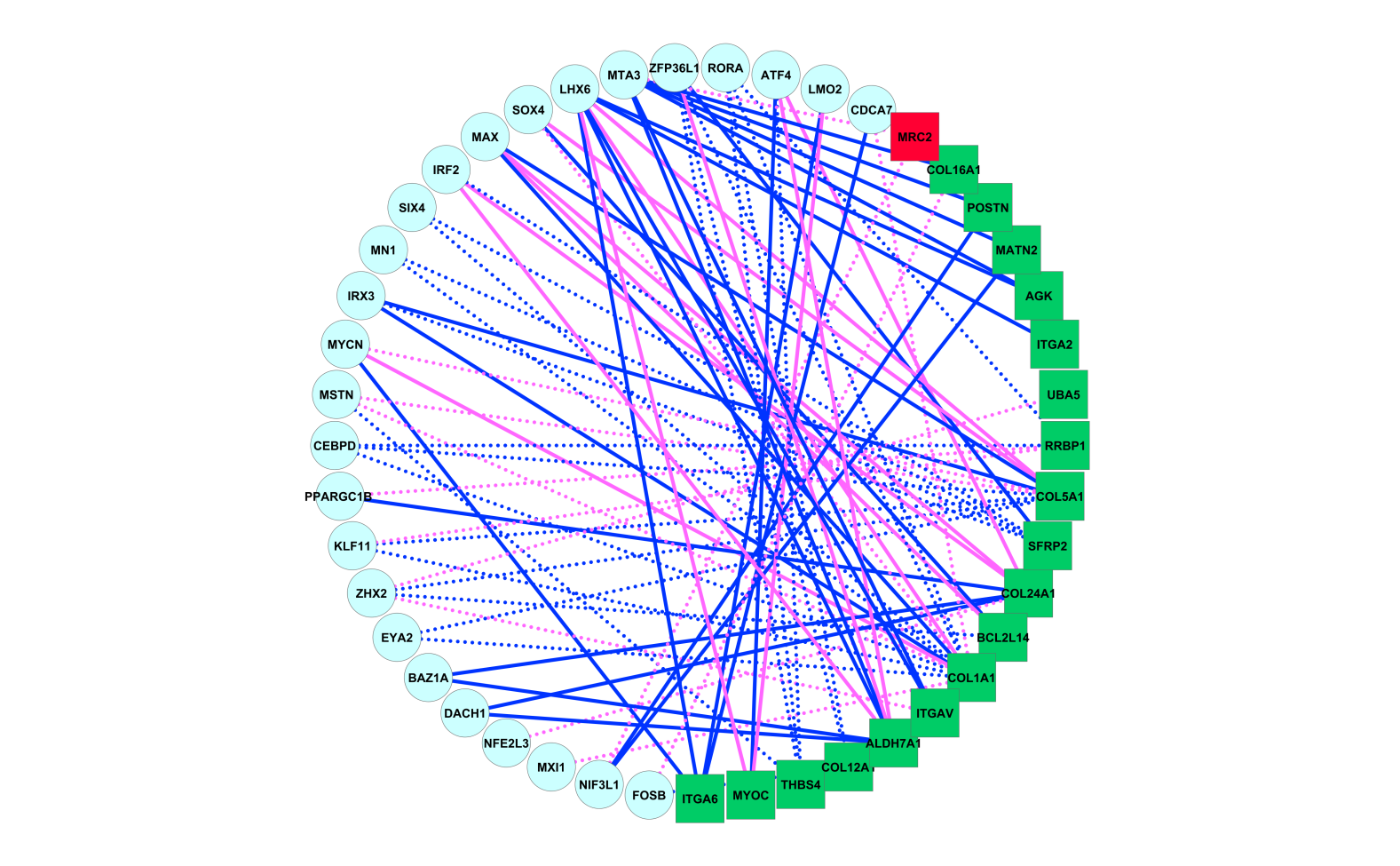

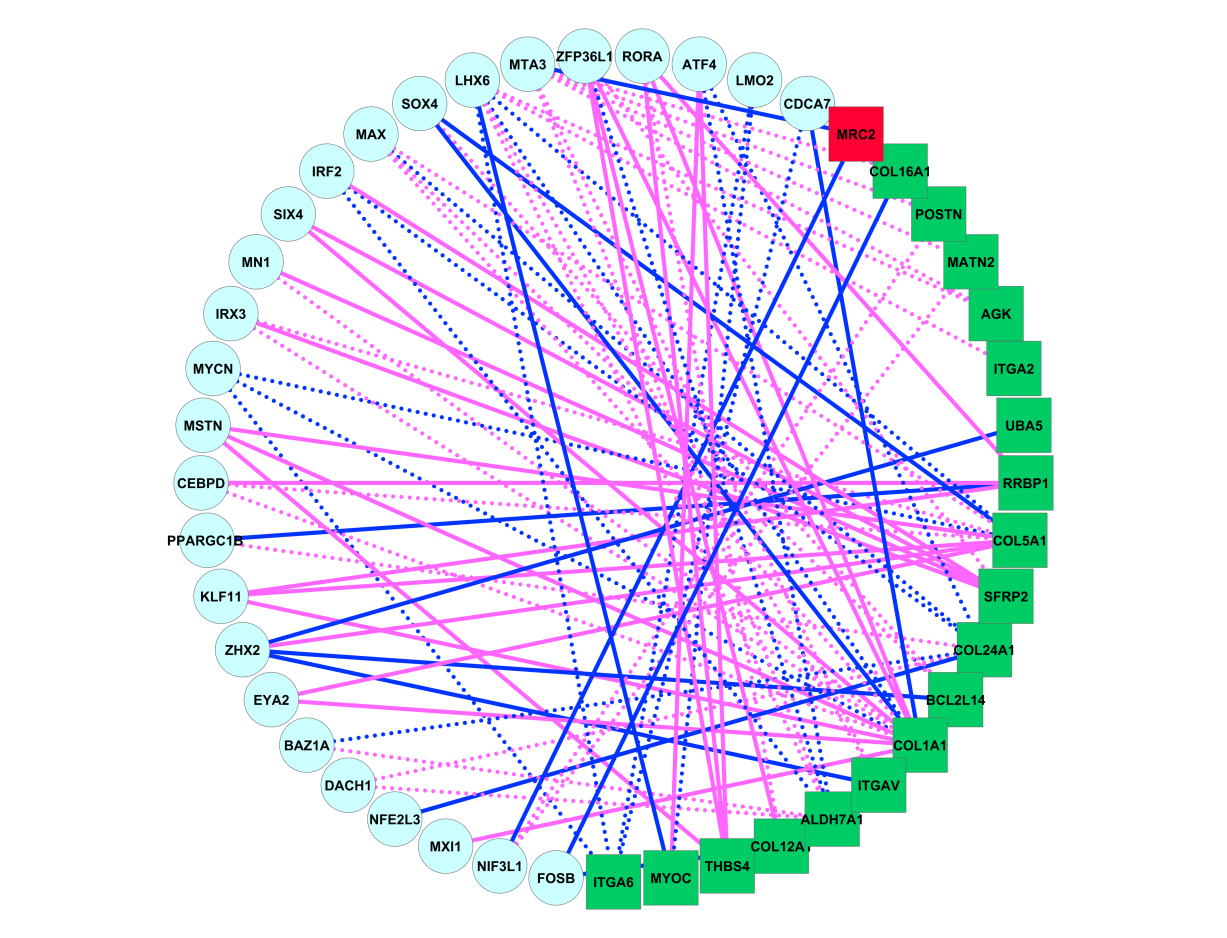

Supplement: Supplementary file 5 — Additional file 5: Graphical representation of the correlations RF-DE genes in network 2 (Connective Tissue Disorders, Dermatological Diseases and Conditions, Cellular Assembly and Organization). Gene expression correlations between RIF-predicted regulatory factors and DE genes, which were significantly different in both genetic types, were graphically represented with Cytoscape 2.8.0, for each genetic type. Light circle nodes represent regulatory factors. Squared nodes are DE genes, red ones are upregulated in IB while green ones are upregulated in DUxIB. Solid lines are significant correlations and dotted lines are non-significantly different from cero (in each genetic type). Blue lines are positive correlations and pink ones are negative correlations. (DOCX 785 KB) [file 12864_2013_6121_MOESM5_ESM.docx]
